# Self-Nanoemulsifying Drug Delivery System (SNEDDS) for Improved Oral Bioavailability of Chlorpromazine: In Vitro and In Vivo Evaluation

**DOI:** 10.3390/medicina55050210

**Published:** 2019-05-24

**Authors:** Jeand Baloch, Muhammad Farhan Sohail, Hafiz Shaib Sarwar, Maria Hassan Kiani, Gul Majid Khan, Sarwat Jahan, Muhammad Rafay, Muhammad Tausif Chaudhry, Masoom Yasinzai, Gul Shahnaz

**Affiliations:** 1 Sulaiman Bin Abdullah Aba Al-Khail—Centre for Interdisciplinary Research in Basic Science (SA-CIRBS), International Islamic University, Islamabad 44000, Pakistan; jeand_baloch@yahoo.com (J.B.); rector@iiu.edu.pk (M.Y.); 2 Riphah Institute of Pharmaceutical Sciences (RIPS), Lahore Campus, Riphah International University, Lahore 54770, Pakistan; farmacist@gmail.com (M.F.S.); h_shoaibsarwar@hotmail.com (H.S.S.); 3 Department of Pharmacy, Faculty of Biological Sciences, Quaid-i-Azam University, Islamabad 45320, Pakistan; marria.h.kyani@gmail.com (M.H.K.); gmkhan@qau.edu.pk (G.M.K.); 4 Department of Animal Sciences, Quaid-i- Azam University, Islamabad 45320, Pakistan; sjahan@qau.edu.pk; 5 Department of Forester, Range and Wild life management, College of Agriculture and Environmental Sciences, The Islamia University, Bahawalpur 63100, Pakistan; Rafay@iub.edu.pk; 6 Environmental Analytical Lab, NPSL, Pakistan Council of Scientific and Industrial Research (PCSIR), Islamabad 45710, Pakistan; tausif_chaudhry@yahoo.com

**Keywords:** self-nanoemulsifying drug delivery system (SNEDDS), long-chain triglycerides, chlorpromazine, solubility enhancement, pharmacokinetics, oral bioavailability

## Abstract

*Background and Objectives:* Lipid-based self-nanoemulsifying drug delivery systems (SNEDDS) have resurged the eminence of nanoemulsions by modest adjustments and offer many valuable opportunities in drug delivery. Chlorpromazine, an antipsychotic agent with poor aqueous solubility—with extensive first-pass metabolism—can be a suitable candidate for the development of SNEDDS. The current study was designed to develop triglyceride-based SNEDDS of chlorpromazine to achieve improved solubility, stability, and oral bioavailability. *Materials and Methods:* Fifteen SNEDDS formulations of each short, medium, and long chain, triglycerides were synthesized and characterized to achieve optimized formulation. The optimized formulation was characterized for several in vitro and in vivo parameters. *Results:* Particle size, zeta potential, and drug loading of the optimized SNEDDS (LCT_14_) were found to be 178 ± 16, −21.4, and 85.5%, respectively. Long chain triglyceride (LCT_14_) showed a 1.5-fold increased elimination half-life (*p* < 0.01), up to 6-fold increased oral bioavailability, and 1.7-fold decreased plasma clearance rate (*p* < 0.01) compared to a drug suspension. *Conclusion:* The findings suggest that SNEDDS based on long-chain triglycerides (LCT_14_) formulations seem to be a promising alternative for improving the oral bioavailability of chlorpromazine.

## 1. Introduction

Approximately 40–70% of the new therapeutic moieties explored in recent years belong to the biopharmaceutical classification system (BCS) class II or IV, showing poor aqueous solubility that limits their oral administration [1,2]. Among several factors responsible for the low oral bioavailability, one main reason is incomplete dissolution and precipitation of drugs in the gastrointestinal (GI) fluids due to their lipophilic nature. Such poorly soluble therapeutic molecules are promising candidates for the lipid-based drug delivery system which significantly facilitates their oral bioavailability [3,4]. Advancements in nanotechnology have paved the way to finding nano-based delivery systems to overcome these limitations. These systems include polymeric nanoparticles, lipid-based systems (liposomes, solid lipid nanoparticles, nanoemulsion), and noisome. The most popular nano-based strategies for lipophilic drugs include incorporation of drugs in lipid vehicles like oil/surfactant dispersion [5], micro and nanoemulsions [6,7], and self emulsifying drug delivery systems. Self-nanoemulsifying drug delivery systems (SNEDDS) have emerged as a vital strategy for the efficient delivery of drugs with poor aqueous solubility [8,9,10]. SNEDDS are well known for their potential to enhance the solubility and absorption of the lipophilic drugs [11] by increasing the surface area and decreasing the size of oil droplets that are readily digestible and incorporated into mixed micelles that can pass the intestinal lumen [12]. Moreover, increase in trans-cellular permeability has also been reported through SNEDDS as they can increase the lipid fluidity of enterocytes’ membranes and inhibit efflux pumps, resulting in enhanced oral bioavailability. SNEDDS have distinct features—which make them superior to conventional micro and nanoemulsions—including log term stability, patient compliance, palatability, reduction in dose, ease of formulation, and scale-up synthesis. Other features of SNEDDS that enhance oral bioavailability include reducing cytochrome-P450 metabolism in gut enterocytes, increasing lymphatic transport via payer-patches, and protecting against the first pass metabolism [12]. Two types of SNEEDS are reported: liquid SNEDDS (L-SNEDDS) and solid SNEDDS (S-SNEDDS), where solidification is achieved through spry drying, melt granulation, and adsorption on inert solids like microcrystalline cellulose, lactose, and aerosol [1].

**Figure 1:**
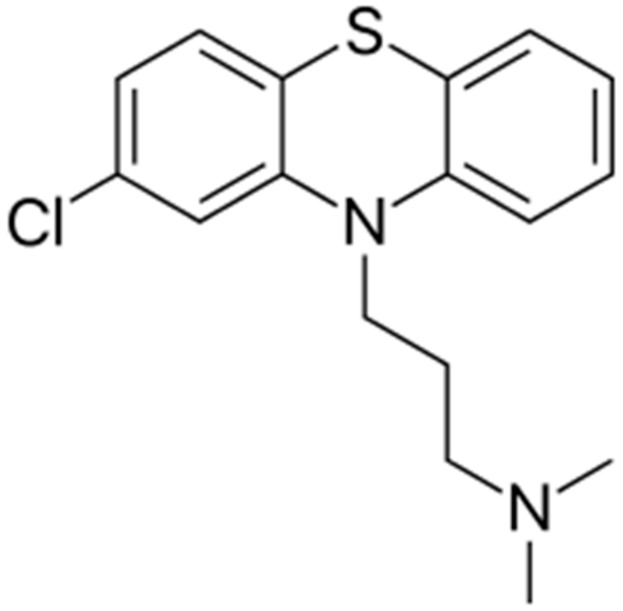
Chemical structure of chlorpromazine.

Chlorpromazine (*log P* = 5.6, aqueous solubility 2.55 mg/L) is one of the most important anti-psychotic and anti-emetic drugs from phenothiazine derivative group [13]. It belongs to biopharmaceutical classification system (BCS) class IV due to its low solubility and permeability. However, chlorpromazine HCl is class III owing to improved solubility yet low permeability [14]. Chemically, it is 3-(2-chloro-10H-phenothiazin-10-yl)-*N*,*N*-dimethyl-1-propanamine (Figure 1), having molecular weight 318.863 g/mol. When administered orally, it is incompletely absorbed with a bioavailability of approximately 4–38% and high protein binding of 90–99% [3]. The time to reach maximum plasma concentration ranges between 1–4 h and 6–24 h following oral and intra-muscular administration, respectively. The exact mechanism of action is still unclear, however, it is believed that chlorpromazine acts as dopamine antagonist variable gastrointestinal absorption and extensive first-pass metabolism are also thought to be responsible for such low oral bioavailability of chlorpromazine [15,16]. Moreover, there is a need to formulate chlorpromazine in such a delivery system that can enhance its bioavailability by increasing solubility, permeability, and decreased first-pass metabolism. Therefore, incorporating chlorpromazine into SNEDDS shall be a promising strategy to enhance its oral bioavailability.

The purpose of this study was to design and develop an optimized SNEDDS formulation of chlorpromazine by using different triglycerides of varying chain lengths to enhance its oral bioavailability. Of these, the selected formulations were evaluated in terms of percentage transmittance, drug content, zeta potential, particle size, physical, chemical and thermodynamic stabilities, followed by dispersibility test. In addition, formulations were evaluated for in vivo oral permeation enhancement of chlorpromazine via chylomicron linked transport through the lymphatic system along with para- and trans-cellular routes, previously reported by our research group [17].

## 2. Materials and Methods 

### 2.1. Material

Chemicals used in the study were purchased from Sigma-Aldrich (Munich, Germany). The solvents used were of HPLC/analytical grade purchased from Merck (Kenilworth, NJ, USA). Chlorpromazine was received from Global Pharmaceutical Pvt. Ltd. Islamabad, Pakistan.

### 2.2. Methods

#### 2.2.1. Determining Drug Solubility 

The solubility of chlorpromazine was determined in all components used for the synthesis of SNEDDS, which include oils (captex, triacetin, linseed oil, and olive oil), surfactants (tween 85), and ethanol. The drug was taken in a stoppered glass vial of 5 mL capacity and mixed for 10 min with each component by using a vortex mixer (China). The vials were kept in an isothermal shaker (GFL1092, Burgwedel, Germany) at 50 ± 1.0 °C for 72 h until homogeneity is achieved. The homogenate was then centrifuged at 3,000 rpm for 10 min to remove the insoluble drug. The supernatants were filtered with 0.45 μ syringe filter and drug concentration was determined through HPLC (Agilent Technologies, Inc., Santa Clara, CA, USA) method reported earlier with following conditions [16]. Separation of the drug was carried out through C_8_ column (ZORBAX Eclipse XDB Agilent Technologies, Santa Clara, CA, USA) by injecting 20 µL sample, using mobile phase acetonitrile and methanol (10:90, v/v) with a flow rate of 1.0 mL/min at 35 °C. Run time was fixed at 6 min and absorbance was measured at 308 nm.

#### 2.2.2. Synthesis of SNEDDS

Each formulation with a total weight of 1 g was prepared by taking 20 mg of drug in Teflon lined screw-capped glass vial and then adding various proportions of glycerides and surfactants. The drug was dissolved in components by gentle stirring and heating at 50 °C in a water bath. The mixture was cooled down to room temperature followed by addition of ethanol and stirring to achieve uniformity. Formulations were kept at ambient temperature for 48 h to achieve the equilibrium and observed for any phase separation and turbidity prior to emulsification and particle size determination. Formulations with no phase separation were selected for stability testing and further characterization.

#### 2.2.3. Dispersibility and Stability Investigations

The efficiency and dispersibility of self-emulsification were determined through USP dissolution apparatus 2. Briefly, 1 mL of each formulation was added dropwise into 200 mL of simulated intestinal fluid (pH 6.8, without enzymes), maintained at 37 °C, with gentle stirring using stainless steel paddles rotated at 60 rpm. Each formulation was assessed visually for the rate of emulsification, dispersibility, apparent physical stability, and appearance. The precipitation of the drug was evaluated after 24 h. The formulations were further categorized as stable (no precipitation), milky, dull white, whitish, or unstable (showing precipitation). The stable formulation with small particles size that passed the dispersibility test was selected for further characterization. 

#### 2.2.4. Drug Content Determination 

The selected formulations were evaluated for drug entrapment. Extraction of the drug from SNEDDS was carried out by taking one part of each formulation and diluting it with nine parts of 100% methanol (v/v) and centrifuged at 10,000 rpm for 30 min. The supernatant obtained was then diluted with methanol (2.5 times) and drug content was determined through HPLC using the earlier reported conditions.

#### 2.2.5. Thermodynamic Stability Profile

Thermodynamic stability studies were carried out for the selected formulations. Nanoemulsions were subjected to centrifugation at 18,000 rpm at 4 °C for 30 min. The stable formulations with no phase separation were further subjected to 6 heating and cooling cycles by incubating them for 48 h at 45 °C and 4 °C, respectively. The formulations that remained stable at former conditions were proceeded to 3 freeze–thaw cycles between −21 °C and 25 °C, and monitored for the time-dependent physical changes like drug precipitation. HPLC analysis for chlorpromazine was carried out to check the chemical stability of drug within SNEDDS. Moreover, selected formulations were kept for three months at 37 ± 2 °C and refrigerator (2–8 °C) to check their stability upon storage and shelf-life. The stability was measured in terms of the change in particle size, dispersibility, and transmittance.

#### 2.2.6. Percentage Transmittance

The percentage transmittance of the SNEDDS gave an idea about the formulation features including uniformity and size of the droplets. Percentage transmittance was measured by taking 1 mL of each formulation and diluting it 10 times with distilled water. A UV spectrophotometer was used to measure the percentage transmittance at 308 nm by taking distilled water as a blank. 

#### 2.2.7. Particle Size and Zeta Potential Analysis

The particles size and zeta potential of the chlorpromazine nanoemulsions were measured through PSS NicompTM 380 DLS/ZLS devise. Furthermore, transmission electron microscopy (TEM) using (FEI Nova NanoSEM 450) was done to examine the surface morphology and particle size of selected SNEDDS formulations. 

#### 2.2.8. Ex Vivo Transport Studies

Ex vivo transport profile of entrapped chlorpromazine from SNEDDS was conducted in simulated gastrointestinal fluid (SIF, pH 6.8) using everted sac method [18]. The study protocol was approved by the Institutional Ethics Committee of Riphah International University, Lahore (REC/RIPS-LHR/2018-018, dated 9 January 2019). Briefly, rats (weighing 250–300 g) were anesthetized using chloroform and abdomen was opened with the middle incision. The small intestine was detached by cutting each end. Middle region of small intestine was taken from the proximal-distal part. The entire length of small intestine was cleaned with saline solution to eradicate blood and debris. The intestine was everted by carefully passing a narrow glass rod from one end of the intestine and then gently rolling it on a glass rod. Ligatures were fixed over the condensed part of the glass rod and exert the sac by softly pushing the rod through the whole length of intestine. The rod was then detached, and the intestine was placed in SIF at room temperature. A 4 cm long piece was tied off with thread and slice an open sac from the main length. Second ligature was positioned loosely around the open end of the sac and a blunt needle was inserted attached with a syringe. The loose ligature was fastened over the needle and 2 mL of chlorpromazine formulation was injected into the sac. The intestine was then placed in 150 mL of SIF (pH 6.8) in a shaking incubator. The samples were collected from the surrounding medium at pre-defined time intervals that was replaced with the same amount of fresh solution. Samples were analyzed using HPLC and percent transport was calculated using the following equation.

Apparent Permeabilty ([μg/cm]^2^) = Concentration × Volume/Mucosal area (1)

Mucosal surface area was calculated by assuming intestine a cylinder and using the formula:

Mucosal surface area cm^2^ = Circumference (πr^2^) × Length (2)

#### 2.2.9. In Vivo Oral Bioavailability Study

Oral bioavailability of the optimized formulations was investigated on male Sprague–Dawley rats weighing 200–250 g. In vivo studies were carried out as per guidelines of approved protocol by Institutional Ethics Committee of Riphah International University, Lahore (REC/RIPS-LHR/2018-018, dated 9 January 2019). Animals were divided into 4 groups (*n* = 6) and housed one day before starting experiment with free access to food and water. The nanoformulations small chain triglyceride (SCT_15_), medium chain triglyceride (MCT_6_) long chain triglyceride (LCT_14_) and chlorpromazine suspension were given orally through gavage at a concentration of 2 mg/kg of body weight of chlorpromazine. The blood samples (200 µL) were collected from the tail vein at predetermined time points of 1, 2, 4, 8, 12, and 24 h in heparinized syringes and centrifuged at 5,000 rpm for 10 min. The plasma was separated, transferred to separate Eppendorf and stored in a freezer at −20 °C until further analysis. The drug was extracted from plasma samples through liquid-liquid extraction by adding 200 µL of chilled acetonitrile and 150 µL of methanol followed by vortex for 5 min and centrifugation at 3,000 rpm for 10 min. The supernatant of each sample was then transferred to labeled HPLC vials and run one by one on HPLC using method described above. Peak areas according to concentration were recorded and a graph between area against time was drawn to calculate plasma drug concentration for all formulations [19]. 

#### 2.2.10. Pharmacokinetic Parameters and Statistical Analysis 

Pharmacokinetic parameters of orally administered chlorpromazine were obtained by using a non-compartments pharmacokinetic analysis of plasma concentration-time data. PK Solver (a free Microsoft Excel Add-in) was used to calculate the area under the curve from concentration versus time curve to last measured time (AUC_0–24_) and other pharmacokinetic parameters. Absolute bioavailability was calculated from absolute dose and areas under curves (AUC) for oral against intravenous administration [20]. 

#### 2.2.11. Statistical Analysis 

Statistical data analysis was performed using Student’s *t*-test with *p* < 0.05 as the minimal level of significance. All values were expressed as mean ± SD. Finally, the results were compared with control and literature.

## 3. Results

### 3.1. Drug Solubility in Nanoemulsion Components 

**Figure 2:**
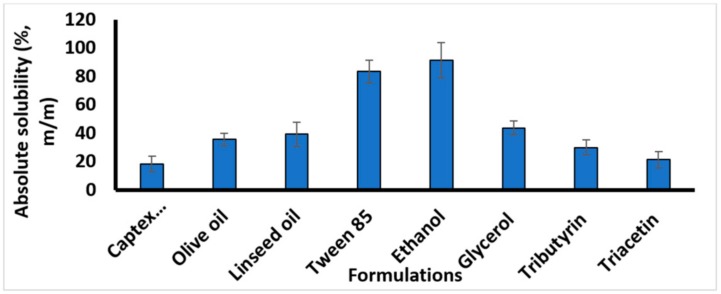
Solubility of Chlorpromazine in different components of self-nanoemulsifying drug delivery systems (SNEDDS). Results are shown as mean ± SD of three different experiments.

The solubility of chlorpromazine was tested in all formulation components individually. Chlorpromazine was found to be more soluble in ethanol (91%) compared to other vehicles as shown in Figure 2. The choice of formulation components was driven by the fact that chosen excipients must have definite regulatory status. Three different types of triglycerides, i.e., long chain, (C18 from linseed and olive oil), medium chain (mono and di-glycerides), and short chain (triacetin, a trimester of glycerol and acetic acid) were chosen. The emulsion is stabilized by the presence of surfactants and co-surfactants, so Tween 85 was added to stabilize the SNEDDS.

### 3.2. Characterization and Evaluation of SNEDDS Formulations

Lipid-based formulations were prepared by using short chain triglycerides (SCT), medium chain triglycerides (MCT) or long-chain triglycerides (LCT). Triacetin was selected as SCT, Captex 355 was used as medium chain triglyceride and olive oil and linseed oil were used as long chain triglycerides. Fifteen different formulations of each of SCT, MCT, and LCT were prepared as SNEDDS by using different ratios of triglycerides, surfactants and co-surfactants as presented in Table 1, Table 2 and Table 3, respectively. These formulations were tested and evaluated to find optimized formulations for further characterization. 

### 3.3. Dispersibility Test

In the formulation of SCT, MCT, and LCT SNEDDS, different concentrations of the oil phase, surfactants and co-surfactants were used, and dispersion time was found to be dependent on composition as shown in Table 1, Table 2 and Table 3. An increase in surfactant to co-surfactant ratio produced smaller particle size and reduced dispersion time. Formulation SCT_15_ (Table 1) has the lowest dispersion time of 35 ± 2 s, which is due to the decreased particle size and greater emulsification ability produced by the highest proportion of surfactant (tween 85). Similar trends were observed in the dispersion time with an increase in the oil phase and surfactant/co surfactants ratio in case of MCT-SNEDDS. However, in comparison to SCT, MCT requires a greater concentration of surfactant (50% tween 85) as presented in Table 2. In the case of LCT formulation combination of linseed oil and olive oil was used. Formulations with higher contents of linseed oil provided greater solubility but the rate of emulsification was compromised due to a proportional decrease in concentration of olive oil. LCT provided optimum emulsification and dispersion time at surfactant (tween 85) concentration of 40% without the use of co-surfactant. However, a further increase in surfactant ratio resulted in precipitation as is evident from results (Table 3).

### 3.4. Stability Tests

Only those formulations that proved their thermodynamic, chemical and physical stability were selected for further studies. The results indicated that formulations SCT_15_, MCT_6_, and LCT_14_ were the most stable formulations (Table 4) from each group of SNEDDS. These formulations were stored for three months at 37 °C ± 2 and refrigerator for stability studies upon storage. Formulations were observed to be more stable in the refrigerator as there was no significant change observed in particle size, poly dispersity, drug loading, percentage transmittance, and dispersibility. Thus, LCT_14_, among the three formulations, was considered to be the most stable nanoemulsion as it showed no absorbance (highest transmittance). 

### 3.5. Zeta potential Analysis 

The zeta potential analysis was carried out on the selected formulation SCT_15_, MCT_6_, and LCT_14_ and respective values are presented in Table 1. The higher the zeta potential, the greater the stability because increased surface charge opposed the aggregation of particles. Zeta potential of SCT_15_, MCT_6_, and LCT_14_ was found to be −17.1, −14.2, and −21.4, respectively. 

### 3.6. Particle Size, Poly Disersity, and Surface Morphology

**Figure 3:**
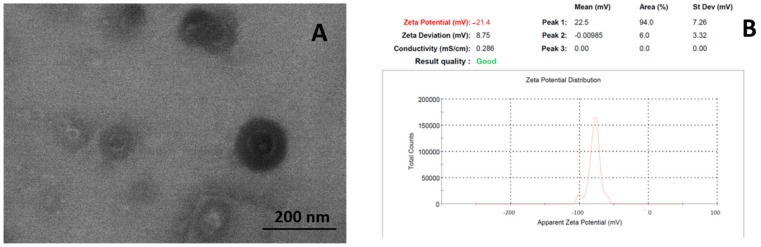
(**A**) Transmission electron micrographs and (**B**) Zeta potential scan of optimized final formulation (LCT_14_).

The selected formulation SCT_15_, MCT_6_, and LCT_14_ were subjected to particle size analysis and the particle size was found to be 159 ± 15, 186 ± 20, and 178 ± 16, respectively (Table 1). The uniformity of the synthesis of SNEDDS was displayed through the polydispersity index (PDI) value. The PDI was found to be 0.27 ± 0.43, 0.13 ± 0.67, and 0.31 ± 0.17 for SCT_15_, MCT_6_, and LCT_14_, respectively. Transmission electron microscopy (TEM) revealed that particles were spherical shaped in case of LCT_14_ SENDDS as compared to SCT_15_ and MCT_6_ (Figure 3).

### 3.7. Drug Content Determination 

**Figure 4:**
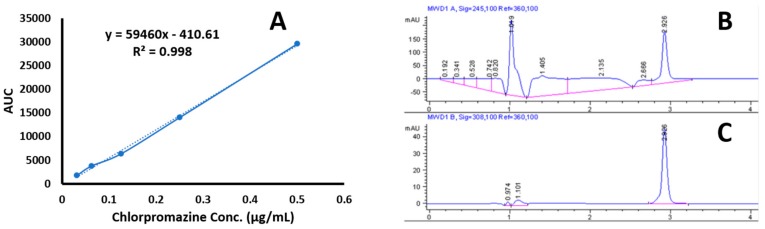
(**A**) Linearity curve of chlorpromazine, (**B**) chlorpromazine chromatogram in formulation, and (**C**) chromatogram of pure chlorpromazine.

Selected formulations SCT_15_, MCT_6_, and LCT_14_ were evaluated by HPLC for estimation of drug content in individual formulations. The aim of this test was to evaluate the formulations for drug loading efficiency. The linearity curve of the chlorpromazine over a range of 0.0312–0.5 µg/mL is shown in Figure 4a. The value of R^2^ was found to be 0.998 with equation Y = 59460 x − 410.61. The chromatogram shown in Figure 4b, presents the chromatogram of chlorpromazine detection in formulation with retention time at 2.296 min. Whereas, Figure 4c shows the chromatogram of pure chlorpromazine with retention time at 2.926 min. A decrease in drug content was observed with an increase in chain length of triglyceride, like 92.3% for SCT_15_, 82.7% for MCT_6_, and 85.5% for LCT_14_ (Table 1). Thus, shorter chain triglycerides showed better encapsulation of drug as compared to LCT.

### 3.8. Ex Vivo Permeation Enhancement

**Figure 5:**
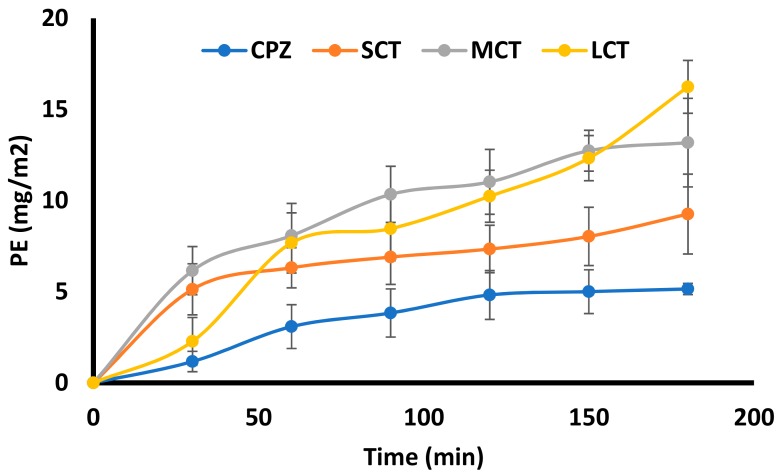
Permeation enhancement of Chlorpromazine (CPZ) from CPZ suspension, CPZ loaded SCT_15_ SNEDDS, CPZ loaded MCT_6_ SNEDDS, and CPZ loaded LCT_14_ SNEDDS across rat intestine through ex vivo studies. CPZ transport expressed as cumulative transport (Mean ± SD, *n* = 3).

The results of ex vivo studies are shown in Figure 5 indicated that drug transport through intestine was gradually increased for formulations as compared to pure chlorpromazine suspension. The highest transport was observed with LCT_14_ SNEDDS resulting in a 3.2 fold increase (16.24 ± 1.45 µg/cm^2^) compared to the chlorpromazine suspension (5.15 ± 0.31 µg/cm^2^) after 3 h. The increase in permeation for SCT_15_ and MCT_6_ SNEDDS was found as 11.18 ± 2.43 µg/cm^2^ and 8.26 ± 2.19 µg/cm^2^, respectively. 

### 3.9. Oral Bioavailability and Pharmacokinetic Studies

**Figure 6:**
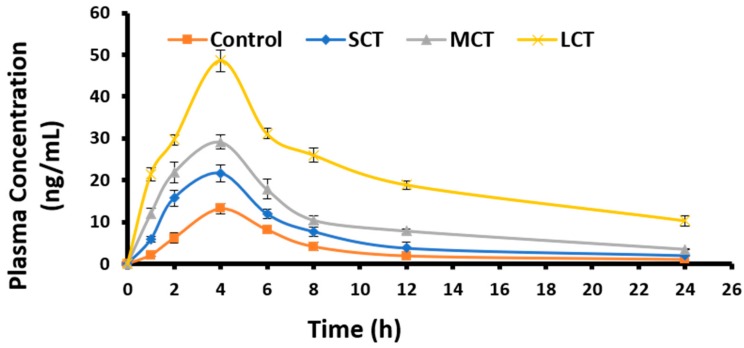
Plasma-concentration of different SNEDDS and chlorpromazine suspension after oral administration in rats (*n* = 6). The samples were taken at predefined time and quantified for chlorpromazine concentration using HPLC. The results are presented as mean ± SD of three different experiments.

Plasma samples obtained from test and control groups of rats (*n* = 6) at predefined intervals were injected to HPLC to analyze the concentration of drug using a validated HPLC method as previously described. The retention time of chlorpromazine was observed to be 2.926 min. Plasma drug concentration was plotted against time to obtain plasma level–time curve (Figure 6) to study the pharmacokinetics of chlorpromazine loaded in SNEDDS. The bioavailability of chlorpromazine SNEDDS was very much higher as compared to the control (Chlorpromazine suspension) with the maximum bioavailability observed in case of LCT_14_. 

The pharmacokinetic study of chlorpromazine for the non-compartmental pharmacokinetic model was done using PK solver. Values for different PK parameters observed for test and control formulations are presented in Table 5. The area under the curve AUC (_0–24_) for LCT_14_ was found to be maximum (525.882 ± 10.815) compared to SCT_15_ (160.491 ± 2.368) and MCT_6_ (253.419 ± 9.721) SNEDDS, while the chlorpromazine suspension showed that the minimum (87.400 ± 1.180). C_max_ was also higher for LCT_14_ (48.642 ± 2.596) compared to MCT_6_ and SCT_15_. Plasma Half-life (t_1/2_) of chlorpromazine was significantly increased with LCT_14_ (9.876 ± 0.251) as compared to chlorpromazine suspension (5.736 ± 0.312). This half-life was highest amongst all SNEDDS formulations. Oral bioavailability increased 6.5-fold compared to chlorpromazine suspension with LCT_14_.

## 4. Discussion 

In the past decade, much attention has been directed to lipid-based formulations with emphasis on improving and enhancing the solubility and oral bioavailability of poorly water-soluble BCS class II drugs. Ideally, SNEDDS transport a hydrophobic drug in solubilized form and retain satisfactory solubilization through gastrointestinal passage. Moreover, SNEDDS protect drugs against enzymatic degradation, foster super saturation, surfactant-provoked membrane fluidity and permeability enhancement that is often sufficient for drug absorption [16]. 

SNEDDS composed of surfactants, co-surfactants, oil, and drug should turn into a monophasic, clear dispersion once added to the aqueous phase, at room temperature. Upon mild agitation in aqueous media SNEDDS were converted to very fine oil/water emulsion. Surfactants used in the formulation are responsible for the conversion of oil phase into the very fine particles by reducing the surface tension at the oil and water interface. The finer the droplet size of oil phase, the lesser the dispersion time of SNEDDS [6]. As the total weight of the SNEDDS formulation was kept constant to 1 g, an increase in the concentration of triacetin produced a proportional increase in the dispersion time due to increased particle size and a simultaneous decrease in the surfactant/co-surfactant ratio [7].

Thermodynamic stability is a distinguishing feature of SNEDDS compared to the simple emulsion formulations that have kinetic stability and become phase separated eventually [4]. These nanoemulsions (SNEDDS) were formed at a specific concentration of oil, surfactants and co-surfactants, and water. Prepared formulations were tested for their thermodynamic stability in terms of permanent phase separation, cracking, coalescence, and creaming by applying freeze–thaw cycles, heating–cooling cycles, and centrifugation. The percentage transmittance of the formulation corresponds to the optical clarity of the formulation as clear dispersion will cause less scattering of incident light compared to opalescent dispersions [7]. 

The triglycerides were selected to observe the effect of solubility as they are easily taken up by the chylomicrons and travel through lymphatic system. SNEDDS were prepared using three different triglycerides. Triacetin was selected as SCT that is triester of glycerol with acetic acid. Captex 355 was used as medium chain triglyceride (C8:C10). Olive oil and linseed oil are used as long chain C-18 triglycerides. Fifteen SNEDDS formulation of each of these SCT, MCT, and LCT were prepared using varying amounts of triglycerides, surfactants and co-surfactants as presented in Table 1, Table 2 and Table 3. Of these, only the formulation from each batch SCT_15_, MCT_6_, and LCT_14_ that showed stability and the required features, was selected. The surface zeta potential plays a significant role in stabilizing the nanoformulations. Generally, the value of zeta potential above 20 is an indicator of stabilized formulation as it results in higher repulsive force between the globules and ruling out the possibility of coalescence [8]. In view of these results, formulation LCT_14_ was found to be the most stable formulation due to inter particulate repulsion allowing them to suspend for a longer period of time [9]. Particle size also plays a key role in drug absorption and distribution. Particles of less than 300 nm are more likely to cross the enterocytes without any difficulty [18]. Particle size depends upon the respective composition of SNEDDS formulation. Increase in proportion of surfactants reduces the interfacial tension and produce smaller droplet size which in turn provides more rapid absorption and greater bioavailability. SCT_15_ produced he smallest droplet sized SNEDDS and the MCT_6_ produced the largest. Comparing all other features of three selected formulations, LCT_14_ was considered to be the optimized and best SNEDD formulation. 

The SNEDDS were designed to study the improvement in chlorpromazine permeability across enterocyte following oral administration. SNEDDS have shown excellent potential to enhance the oral bioavailability of highly lipophilic drugs [21]. Ex vivo permeation studies can mimic the potential improvement in drug permeability once inside the GIT [10]. The results of ex vivo permeation revealed a 3.2-fold increased permeation with LCT_14_ across the rat intestine as compared to other formulations. The results fairly indicate the effect of triglycerides on the opening of tight junctions and facilitating the transport through para-cellular route, resulted in increased permeation across the intestinal mucosa. This further suggests the role of oleic acid as strong mucosal permeation enhancer by opening the tight junction and facilitating the para-cellular transport [12].

Oral pharmacokinetics of SNEDDDS loaded with chlorpromazine were studied in the rat model. It has been previously observed and reported that these SNEDDS, being lipid in nature can enhance synthesis of intestinal chylomicrons, taking a drug through the lymphatic system to systemic circulation along with facilitating drug transport through trans-cellular and para-cellular pathways [13]. The selected formulations SCT_15_, MCT_6_, and LCT_14_ were orally administered to study the change in bioavailability with these formulations as compared to control chlorpromazine suspension. These formulations were found to be stable through different physicochemical parameters like sufficient transmittance, suitable emulsification time and drug loading, reported earlier. SNEDDS provide an excellent lipid source for stimulation of chylomicrons production, which in turn provides increased lymphatic transport of drugs. Lipids are digested into smaller entities, absorbed into enterocytes, and converted to lipids again by re-esterification and are incorporated into intestinal chylomicrons [17]. Furtehrmore, medium chain mono and diglycerides have been reported to interact with tight junction by interacting with tight junction proteins F-actin and ZO-1. A mechanistic study confirmed the opening of tight junction by the use of SNEDDS containing a solubilizer [15]. LCT_14_ showed a more than 1.5-fold increased elimination half-life (*p* < 0.01), 3.7-fold increase in plasma drug concentration (*p* < 0.01) and a 1.7-fold decreased plasma clearance rate (*p* < 0.01) compared to native drug. The absolute oral bioavailability (versus I.V. injection) of LCT_14_ calculated on basis of AUC_0–24_ was about 40% as compared to native drug suspension 6.6%. This significant increase in the bioavailability of highly lipophilic drugs (chlorpromazine) might occur due to the formation of hydrogen bonds with cholesteryl esters and triglyceride, which facilitate their lymphatic transport via chylomicron uptake. According to these investigations, SNEDDS based on long-chain triglycerides (LCT_14_) formulation seems to be a promising carrier to improve the oral bioavailability of chlorpromazine.

## 5. Conclusions

In this study, SNEDDS based on small chain triglycerides (SCT), medium chain triglycerides (MCT), and long-chain triglycerides (LCT) for oral delivery of highly lipophilic drug chlorpromazine, were successfully designed with the significantly superior features based on different component ratios. Among these, LCT_14_ showed greater potential in term of reduced particle size (178 ± 16), high drug loading (85.5%), and increased oral bioavailability. The formulation was stable over a 3-month storage period at 25 °C and 4 °C in terms of particle size, physical appearance, and drug loading. Hence, the present approach demonstrated the substantial increase in oral bioavailability of highly lipophilic drugs through the use of SNEDDS that adopts intestinal lymphatic route along with para- and trans-cellular route.

## Figures and Tables

**Table 1:** Effect of formulation (small chain triglycerides (SCT-SNEDDS)) composition on the dispersibility, precipitation, appearance, and particle size (mean ± S.D., *n* = 3) for the lipophilic drug chlorpromazine when added to dispersion media simulated intestinal fluid (SIF) pH 6.8 (gentle agitation was provided by a dissolution paddle rotating at 60 rpm).

Code	Composition (% w/w)					Dispersion Time	Precipitation	Appearance	Particle Size
	Triacetin	Tween 85	Ethanol	Drug	Glycerol	(s)			nm
SCT_1_	65	20	5	2	8	126 ± 31	Dim white	Unstable	871 ± 24
SCT_2_	60	25	5	2	8	120 ± 23	Dim white	Unstable	758 ± 15
SCT_3_	55	30	5	2	8	118 ± 9	Dim white	Unstable	414 ± 33
SCT_4_	50	35	5	2	8	99 ± 4	Dim white	Unstable	367 ± 27
SCT_5_	45	40	5	2	8	59 ± 2	Dim white	Stable	232 ± 14
SCT_6_	40	45	5	2	8	43 ± 5	Dim white	Stable	243 ± 16
SCT_7_	60	20	5	2	13	98 ± 13	Dim white	Unstable	716 ± 32
SCT_8_	55	25	5	2	13	75 ± 11	Dim white	Unstable	532 ± 38
SCT_9_	50	30	5	2	13	73 ± 8	Dim white	Unstable	365 ± 21
SCT_10_	45	35	5	2	13	60 ± 4	Dim white	Stable	319 ± 25
SCT_11_	40	40	5	2	13	55 ± 7	Dim white	Stable	246 ± 19
SCT_12_	55	33	5	2	5	57 ± 6	Dim white	Unstable	328 ± 39
SCT_13_	50	38	5	2	5	53 ± 5	Dim white	Stable	247 ± 32
SCT_14_	45	43	5	2	5	49 ± 7	Dim white	Stable	181 ± 11
SCT_15_	40	48	5	2	5	30 ± 4	Dim white	Stable	159 ± 15

**Table 2:** Effect of formulation (medium chain triglycerides (MCT-SNEDDS)) composition on the dispersibility, precipitation, appearance, and particle size (mean ± S.D., *n* = 3) for the lipophilic drug chlorpromazine when added to dispersion media simulated intestinal fluid (SIF) pH 6.8 (gentle agitation was provided by a dissolution paddle rotating at 60 rpm).

Code	Composition (% w/w)					Dispersion Time	Precipitation	Appearance	Particle Size
	Captex 355	Tween 85	Ethanol	Drug	Glycerol	s			nm
MCT_1_	60	25	5	2	8	140 ± 20	White	Stable	911 ± 41
MCT_2_	55	30	5	2	8	112 ± 15	White	Unstable	921 ± 18
MCT_3_	50	35	5	2	8	98 ± 26	White	Unstable	522 ± 21
MCT_4_	45	40	5	2	8	74 ± 27	White	Stable	531 ± 34
MCT_5_	40	45	5	2	8	67 ± 19	White	Unstable	238 ± 28
MCT_6_	35	50	5	2	8	17 ± 4	White	Stable	186 ± 20
MCT_7_	55	25	5	2	13	109 ± 31	White	Unstable	915 ± 19
MCT_8_	50	30	5	2	13	83 ± 18	White	Unstable	732 ± 43
MCT_9_	45	35	5	2	13	76 ± 25	White	Unstable	554 ± 32
MCT_10_	40	40	5	2	13	63 ± 17	White	Unstable	428 ± 29
MCT_11_	35	45	5	2	13	41 ± 12	White	Stable	334 ± 21
MCT_12_	50	33	5	2	10	77 ± 10	White	Unstable	919 ± 17
MCT_13_	45	38	5	2	10	66 ± 11	White	Unstable	465 ± 18
MCT_14_	40	43	5	2	10	43 ± 16	White	Stable	365 ± 38
MCT_15_	35	48	5	2	10	37 ± 17	White	Stable	219 ± 18

**Table 3:** Effect of formulation (long chain triglycerides (LCT-SNEDDS)) composition on the dispersibility, precipitation, appearance, and particle size (mean ± S.D., *n* = 3) for the lipophilic drug chlorpromazine when added to dispersion media simulated intestinal fluid (SIF) pH 6.8 (gentle agitation was provided by a dissolution paddle rotating at 60 rpm).

Code	Composition (% w/w)				Dispersion Time (s)	Appearance	Precipitation	Particle Size (nm)
	Olive Oil Linseed Oil (1:2 w/w)	Tween 85	Ethanol	Drug				
LCT_1_	70	20	8	2	153 ± 36	Milky	Unstable	939 ± 75
LCT_2_	65	25	8	2	139 ± 36	Milky	Unstable	869 ± 123
LCT_3_	60	30	8	2	97 ± 18	Milky	Unstable	791 ± 52
LCT_4_	55	35	8	2	58 ± 13	Milky	Stable	282 ± 24
LCT_5_	50	40	8	2	36 ± 20	Milky	Unstable	234 ± 85
LCT_6_	45	45	8	2	98 ± 36	Milky	Unstable	592 ± 152
LCT_7_	65	28	5	2	137 ± 23	Milky	Unstable	806 ± 74
LCT_8_	60	33	5	2	72 ± 14	Milky	Unstable	386 ± 118
LCT_9_	55	38	5	2	93 ± 10	Milky	Unstable	341 ± 64
LCT_10_	50	43	5	2	84 ± 21	Milky	Stable	297 ± 29
LCT_11_	45	48	5	2	57 ± 17	Milky	Stable	518 ± 38
LCT_12_	65	30	3	2	63 ± 24	Milky	Unstable	231 ± 56
LCT_13_	60	35	3	2	58 ± 19	Milky	Stable	229 ± 19
LCT_14_	55	40	3	2	22 ± 6	Milky	Stable	178 ± 16
LCT_15_	50	45	3	2	87 ± 15	Milky	Unstable	721 ± 182

**Table 4:** Results of physico–chemical tests performed on selected formulations from each of small chain triglyceride (SCT), medium chain triglyceride (MCT), and long chain triglyceride (LCT) self-nanoemulsifying drug delivery system (SNEDDS) loaded with chlorpromazine.

Formulation	Thermodynamic Stability	Drug Loading (%)	Zeta Potential	Particle Size (nm)	PDI	Transmittance (%)	pH
Small chain triglyceride (SCT_15_)	Stable	92.3	−17.1	159 ± 15	0.27 ± 0.43	1.5	7.3 ± 1.6
Medium chain triglyceride (MCT_6_)	Stable	82.7	−14.2	186 ± 20	0.33 ± 0.67	0.1	7.3 ± 1.52
Long chain triglyceride (LCT_14_)	Stable	85.5	−21.4	178 ± 16	0.31 ± 0.17	0.0	7.4 ± 1.0

**Table 5:** Pharmacokinetic studies of orally administered SCT, MCT, and LCT SNEDDS loaded with Chlorpromazine. Results are presented as mean ± SD of different experimental groups of rabbits (*n* = 5).

Pharmacokinetic Parameter	Control	SCT	MCT	LCT
AUC _0 – t_ (µg/mL/h)	87.400 ± 1.180	160.491 ± 2.368	253.419 ± 9.721	525.882 ± 10.815
t_1/2_ (h)	5.736 ± 0.312	6.195 ± 0.154	7.177 ± 0.094	9.876 ± 0.251
K_d_ (h^-1^)	0.121 ± 0.006	0.111 ± 0.002	0.096 ± 0.001	0.070 ± 0.008
K_a_ (h^-1^)	0.548 ± 0.172	1.166 ± 0.192	1.150 ± 0.290	0.633 ± 0.081
C_max_ (µg/mL)	13.165 ± 1.454	21.595 ± 0.978	29.134 ± 1.62	48.642 ± 2.596
F_rel_ (%)	6.6%	12%	19%	40%

AUC 0 – t plasma drug concentration over time interval 0-t h, t1/2 – half-life, Kd – drug disposition rate constant, Ka – absorption rate constant, Cmax – maximum serum concentration that a drug achieves in a specified compartment or test area of the body after the drug has been administrated and before the administration of a second dose, Frel - relative bioavailability.
